# A Qualified Multilingual Assessment for Medical Students: A Cohort Study

**DOI:** 10.7759/cureus.103433

**Published:** 2026-02-11

**Authors:** Nayantara Biswas, Gavin Warner, Marcie Naumowicz, Kari E Hannibal, Jennifer Kasper, Jeffrey Katz, Rose L Molina

**Affiliations:** 1 Obstetrics and Gynecology, Beth Israel Deaconess Medical Center, Harvard Medical School, Boston, USA; 2 Office of Scholarly Engagement, Harvard Medical School, Boston, USA; 3 Pediatrics, Brigham and Women's Hospital, Harvard Medical School, Boston, USA; 4 Orthopedic and Arthritis Center for Outcomes Research, Brigham and Women's Hospital, Boston, USA

**Keywords:** clinical communication, language assessment, language concordant care, medical education, patient safety

## Abstract

Introduction

Effective clinical communication is essential for delivering high‑quality care and directly impacts the safety of patients who prefer a non‑English language. However, no national standard exists for assessing oral non-English language skills among multilingual medical students in the United States.

Methods

From June 2024 to November 2025, medical students who self-reported “very good” or “excellent” oral proficiency in a non-English language were invited to complete the ALTA Speaking and Listening Assessment, a qualified multilingual assessment (QMA) used to verify skills for direct communication in clinical care for patients with non-English language preference. Surveys captured students’ perceptions of the assessment and use of non-English languages with patients after their clinical rotations. We present descriptive statistics of quantitative data and content summaries of qualitative data.

Results

There were 92 eligible students who completed 102 ALTA exams. Of these, 81 students passed at least one assessment. Most participants rated the assessment as extremely or very accurate (n=28, 82.3%) and the scheduling process as very or extremely easy to schedule (n=35, 97.2%). Some found the assessment overly formal. Among the 24 students who completed clinical rotations, 66.7% were asked to interpret, and 50% regularly used a non-English language with patients. Students described increased confidence and rapport-building with patients.

Conclusion

The QMA was feasible and acceptable for medical students. More research is needed to differentiate skills for direct language-concordant care from medical interpretation.

## Introduction

Language access through certified medical interpreters or qualified multilingual healthcare workers is critical for effective communication, patient safety, and trust between clinicians and patients [1]. Integrating language access training into undergraduate medical education is a growing area of curricular development [2]. Such curricula prepare future clinicians to recognize when and how to work with interpreters effectively and understand the structural barriers patients with non-dominant language preferences face in the health care system [3,4].

In the United States (US), there is no national standard for assessing non-English oral language skills for medical professionals, despite increasing regulatory imperatives and health equity incentives for meaningful language access through qualified language-concordant clinicians or professional medical interpreters for patients with non-English language preference [4]. The Department of Justice warns against relying on self-reported language ability and supports independently administered language assessments for federally funded programs [1,5].

Although hospitals and health systems are beginning to implement qualified multilingual assessments (QMAs) to verify clinicians’ self-reported non-English language preferences for direct patient communication (not medical interpretation, which requires a separate assessment), these initiatives typically exclude medical students [6]. The QMA is a formal process used to verify that an individual possesses the requisite linguistic proficiency and cultural responsiveness to provide safe, direct care to patients in a language other than English without the need for an interpreter [7]. Most medical schools rely on self-assessment of students’ language proficiency. In practice, many of these students find themselves serving as informal language intermediaries through interpreting, clarifying instructions, or facilitating rapport with patients whose preferred language is not English [7]. Such informal interpretation carries risks related to patient safety due to miscommunication. To address this challenge, we created a novel QMA policy for medical students and conducted an assessment of its feasibility and acceptability. The primary objective of the study was to evaluate the feasibility and acceptability of a standardized oral language assessment across multilingual medical students. Secondary objectives included characterizing students’ experiences using their non-English language skills during clinical rotations and identifying opportunities for improvement.

## Materials and methods

We led a cohort study of medical students to 1) assess their experiences with taking the ALTA Speaking and Listening Assessment (the QMA exam used at hospitals affiliated with the medical school) and 2) describe their experiences in using non-English language skills with patients during clinical rotations. The ALTA Speaking and Listening Assessment is a commercially available oral evaluation to verify language competency for direct patient care, not interpretation. Assessments are available in more than 80 languages and are generally short (30-90 mins), with prices varying from $60 to $120 per assessment. A 12-point rubric summarizes conversational skills [8]. We selected this language assessment because it was already approved and implemented at affiliated hospitals.

We launched the QMA pilot in June 2024 after obtaining approval for the QMA policy from the Educational Policy and Curriculum Committee, the medical school’s governing body for educational standards. We designed the QMA policy based on a recent national Delphi consensus study [7]. We offered the ALTA Speaking and Listening Assessment to any medical student who self-reported “very good” or “excellent” oral proficiency in a non-English language based on the Interagency Language Roundtable-Healthcare scale [9]. This publicly available instrument was originally validated as the Interagency Language Roundtable (ILR) for physicians and later renamed the ILR for healthcare to encompass a broader range of clinical cadres [10,11]. Students were permitted to take the ALTA assessment in any of the languages that were offered and could test in multiple languages, as long as they self-reported their oral language proficiency as either “very good” or “excellent”. Once a student opted into taking the ALTA assessment, the Medical Language Program administrator sent instructions for scheduling the exam. The administrator received all scores within 1-2 weeks and sent each student an official letter with their results. The survey questionnaires for the post-ALTA and post-PCE surveys are included in the Appendix. If the student received a passing score (minimum score of 9 out of 12), the student could pick up a button that indicated that they are qualified to speak a non-English language for patient care, serving as a visible sign to patients and clinical staff, and providing students with confidence to use their language skills in clinical settings (see the Appendix).

Students were asked to complete a six-item survey about their experiences with scheduling the assessment and their perceptions of the assessment accuracy in evaluating their language proficiency. Once the students completed their principal clinical experience (PCE) (the core year of required clinical rotations), they became eligible for a follow-up survey, which included eight items about their experiences with using the non-English language with patients on clinical rotations and the frequency of wearing the language button. Patients self-identified their preferred spoken language and disclosed this to a medical student who spoke their preferred language. The survey instrument underwent face validation through cognitive debriefing interviews with three members of the Medical Language Program to refine the questions for clarity and content. Feasibility was defined as the ease of scheduling and completing the QMA. Acceptability was defined as the number of eligible students who actually completed the QMA.

We report results from the first 1.5 years of QMA implementation between June 2024 and November 2025. We defined feasibility as the extent to which the QMA was actually taken. We defined acceptability as the extent to which students had positive experiences with taking the QMA. We analyzed anonymous survey data from students who completed the ALTA assessment. We report descriptive statistics of the ease of scheduling, perceived accuracy of the assessment, and frequency of using the target language. Because the surveys were anonymous, we do not have paired individual responses at the two time points. Instead, we used language as a linking variable to compare aggregate response patterns and trends across the two survey time points. The principal investigator applied content analysis to the free-text comments about student experiences with the ALTA assessment, using inductive coding, which was reviewed and discussed with the first author. There were no discrepancies between the reviewers to resolve during this process. Given the brevity of the comments and the small number of responses, we conducted a rapid qualitative analysis of the QMA's features to describe students' experiences rather than to develop an overarching theory.

The HMS Program in Medical Education’s Educational Scholarship Review Committee determined this study as educational quality improvement and did not require IRB review. Data were analyzed and descriptive statistics were calculated using Microsoft Excel. Data visualizations and figures were generated using R statistical software.

## Results

There were 92 students who reported having “very good” or “excellent” oral proficiency in a non-English language and signed up to take the ALTA exam. Among those 92 students, 102 ALTA exams were taken (some students tested in more than one non-English language). Of the 102 assessments, 89 (87.3%) met the passing threshold among 81 students. Spanish and Mandarin were the most frequently assessed languages, with 15 (41.7%) students testing in Spanish and 13 (36.1%) students testing in Mandarin (Table 1). Six students (16.7%) took the test in multiple languages. The majority of students rated the test as very accurate (n=18, 52.9%) or extremely accurate (n=10, 29.4%) in assessing their language skills.

**Table 1:** Summary Statistics for Post-ALTA Assessment and Post-PCE Surveys PCE: Principal Clinical Experience

	N	%
Post-ALTA Assessment Survey (N=36)		
Language(s) Assessed		
Spanish	15	41.7%
Mandarin	13	36.1%
Korean	3	8.3%
Cantonese	2	5.6%
Hindi	2	5.6%
Tamil	2	5.6%
Arabic	1	2.8%
Portuguese	1	2.8%
Ease of scheduling ALTA		
Extremely easy	23	63.9%
Very easy	12	33.3%
Somewhat easy	1	2.8%
Assessment taken in multiple languages		
No	30	83.3%
Yes	6	16.7%
Accuracy of ALTA (N=34)		
Extremely accurate	10	29.4%
Very accurate	18	52.9%
Somewhat accurate	5	14.7%
Not very accurate	1	2.9%
Post-PCE Survey (N=24)		
Language(s) Spoken		
Spanish	9	37.5%
Mandarin	6	25.0%
Korean	4	16.7%
Arabic	2	8.3%
Cantonese	2	8.3%
Tamil	2	8.3%
Farsi	1	4.2%
French	1	4.2%
Portuguese	1	4.2%
Romanian	1	4.2%
Frequency of wearing the language button		
Always	1	4.2%
Very frequently	3	12.5%
Somewhat frequently	4	16.7%
Rarely	6	25.0%
Never	10	41.7%
Frequency of speaking non-English language with patients		
Multiple times per week	6	25.0%
Multiple times per month	6	25.0%
Once per month	4	16.7%
Less than once per month	6	25.0%
Never	2	8.3%
Asked to interpret for others on medical team		
Yes	16	66.7%
No	8	33.3%

Students valued the opportunity to demonstrate their language skills and thought the ALTA assessment was appropriate for their purpose and level (Table 2). While most students reported positive experiences, some expressed concerns about the test format, which did not reflect authentic clinical encounters with patients. Several medical students also felt that the test included unfamiliar medical phrases and that they wished they had more formal preparation before taking the test.

**Table 2:** Content Analysis of Free-Text Survey Responses * Text redacted for anonymity

Post-ALTA Assessment Survey (Themes and Quotes)
ALTA assessment was at the appropriate level for evaluating language proficiency
I thought it was quite challenging, but appropriately so, even though I speak fluently with my parents. In particular, it's a good assessment of medical language proficiency and not just ability to converse conversationally in the language tested.
It was appropriately difficult.
I thought it was a fair assessment of my medical language skills.
It was difficult but fair!
I thought it was very comprehensive, and it was easy to understand the task.
Very fair and helpful
It's a useful test and appropriate for medical students, despite their limited clinical knowledge.
Positive experiences
The rep told me the interview won't be too medically focused and will test me on conversational vocabularies mainly but my interview was very medically focused, requiring me to know some complicated medical conditions in *. Also my tester thought I was a nurse the whole time when I introduced myself as a medical student. I'm not sure if that was reflected on their evaluation of me.
Very cool to have physical, quantifiable proof of language proficiency and for patients to get to see it on my white coat! But at the same time doesn’t require the level of time/commitment as getting medical interpreter certified.
Very useful
It was very straightforward, simple to do, and fast.
I found it accessible and easy to navigate.
Actually, a lot of fun to do the spoken exam
Easy to do
Very straightforward and rapid response about whether we passed or not.
Format may not reflect clinical encounters with patients
Currently, the language courses do not teach to pass ALTA, and there doesn't seem to be support if a student wants to take ALTA but is not quite at the level to pass
It felt like an accurate representation of my language skills, but the question format wasn't what I expected. The style of question was akin to an interview (e.g. how the relationship between doctors and nurses has changed in the last decade) than an actual patient interaction or vocabulary test. I wish I had known to expect an interview (for some of the questions I was like, I don't have coherent thoughts about this in English, let alone Spanish).
It was very formal and may not be representative of how people actually speak * today. * is considered one of the oldest living languages so the assessment was similar to what it would be like if I assessed someone's English proficiency based on their ability to understand and speak Shakespearean English.
Overall, I thought it was a fair test. I do think it misses some of the real-world situations that are key to communication - for instance, there is a difference between not knowing a word but realizing that the word is unknown to you (and thus asking for clarification from your patient) vs completely misunderstanding a word. The one-way nature of the test does not allow for that type of clarification to take place and so one cannot differentiate between these two situations.
It was straightforward; however, answering the questions required more medical knowledge than I have (* student)
* can be spoken in formal or in dialect. Almost all * understand my dialect, but the examiner kept making me speak in formal. So not necessarily an issue but maybe a slightly unnecessary rule.
Unfamiliar medical phrases
I thought it was harder than I anticipated just because it involved the use of a lot of medical language that I don't normally use despite being fluent in my second language and speaking it with my family.
The test included certain medical phrases that were unfamiliar to me as a Native speaker, nevertheless, I was graded fairly and my score allows me to communicate with patients, which is great!
More preparation needed
Format was a little unclear before taking it, perhaps HMS could communicate the format better/gather testimonials from students
Currently the language courses do not teach to pass ALTA, and there doesn't seem to be support if a student wants to take ALTA but is not quite at the level to pass
There wasn't much time to answer questions and it felt awkward to answer medical questions as an M1 student. Overall, the system worked well.
Post-PCE Survey (Themes and Quotes)
Positive experiences with QMA buttons
Positively
They loved it!
Faculty appreciated it. It relieved their concerns about having me as a translator.
They felt comfortable they know someone who they can speak freely
Appreciated and acknowledged bilingual abilities
I usually inform teams that I speak the language before we enter the room of a patient whose main language is not English, and if they're alright with me conversing with the patient, it has worked quite well. I have only used it for Spanish extensively, and a couple times for * with * speaking patients.
Very well
Patients appreciated it
Did not use QMA button
Did not use
I have not had the chance to wear it, as I am not participating in PCE.
I did not use the button, but I wore the (hospital) translator badge everyday that was provided by passing the ALTA language assessment.
I got the button after being done with PCE and most of my other rotations.
When I was able to pick up the pin, I only had one clinical rotation left and it was in the ICU. In the ICU, most patients were intubated/sedated and unable to speak to me so I couldn't really assess the utility of the pin given I only had one rotation left to use it.
N/A to the above question, and PCE already complete
Did not get the button
Did not wear
Unfortunately, I am currently in my PhD years and I have not had the chance to use it in clinical settings
I did not wear the button because I had already completed PCE by the time I took the exam
Positive patient experiences with bilingual student
I have had successful patient encounters in Spanish! It has been great!
My patients appreciated having someone on their team who spoke their native language.
Patients usually appreciated it because they felt more comfortable and relatable.
Patients are very happy to have a provider who speaks the same language as them, as it makes their appointment go much smoother
Worked well
They felt comfortable knowing they can trust someone and share their concerns
It seems like they're able to approach me more easily
Individuals were more receptive and assumed medical proficiency in second language
Experiences have generally been quite good
I've seen relief on patients' faces that someone on their medical team can speak their language fluently. It has been super rewarding to be able to use Spanish with patients!
Made patients feel more comfortable speaking their native language in front of me and allowed me the chance to translate a bit when there was a slight block in understanding of their mechanism of disease
I honestly love it! very grateful
Patients were surprised that I speak * fluently given the fact that it’s usually difficult to find * speaking providers
Positive clinical team experiences with bilingual student
They have been happy to have a Spanish speaking member on the team!
My health professional colleagues appreciated having someone on their team who could communicate with non-English-speaking patients
They appreciated it because it could be hard to get an interpreter on time during rounds sometimes. They felt more comfortable asking me to interpret when I wear the button. It also relived their concerns on exploiting medical students as interpreters. They also appreciated the special connection I could make with patients.
I have used my skills to help translate for supervisors and colleagues
Supervisors are also very appreciative, as it takes away the necessity to use an iPad for translation
Many colleagues and supervisors commented that it was useful to have an interpretation badge, despite being in a language that does not commonly require interpretation in the hospital
It has helped rounds go more smoothly than if we were to use an electronic interpreter
Attendings have been very happy to have someone on the team speak Spanish. I've never had an attending ask me to interpret for them.
Faculty and supervisors appreciated it think it is a great service to the patients
Student agreed to interpret when asked
I accepted it
I would agree because I feel comfortable in my native languages.
I said yes
I am happy to assist in interpreting
Positively
I was excited but also a little nervous not to make any mistakes.
I always communicated that I am not allowed to interpret, but some faculty still convince me to act as an interpreter.
Enthusiastically
I did it
Well
I will notify them the limitations of what I can do as a bilingual provider to draw boundaries but I would kindly help them interpret
Very well
I respond by explaining to the pt that the button doesn’t mean that I am a certified translator; however, I’m fluent in * and would be happy to assist in translating.
I would interpret if the scenario was "lower-stakes" (for example, I would interpret if it was to inform the medical team on how the patient was feeling, or to pass along patient questions to the medical team, but I would not interpret to get consent for a medical procedure).
Student did not agree to interpret when asked
I explained that I am not qualified to interpret and asked them to call an interpreter.
I always communicated that I am not allowed to interpret, but some faculty still convince me to act as an interpreter.
Though I believe I am technically not allowed to interpret and only allowed to speak to patients for my own encounters, I often felt that my medical interpreting skills were stronger and more efficient than that of most of the iPad interpreters. When I would not be asked to interpret, some of the interpreters would often use slightly incorrect terminology, or there might be significant lags in the conversation, that sometimes made the conversations even harder to navigate.
Language not needed in patient care
I am a student at * so, the number of pts I see in the dental clinic is pretty small compared to the number of pts seen at the *-affiliated hospitals. I thus have never come across a patient who has needed any * translation. I do have 1 patient who speaks *, but they speak English as their first language, so translation services are not required. I thus, do not wear the language button since I already know my patients and the languages they speak. If any of my classmates have a *-speaking patient who requires translation, they know they can reach out to me. However, this has not been necessary in the past year.
N/A, and unfortunately I have not met any * patients at all throughout the PCE and post-PCE period

**Figure 1:**
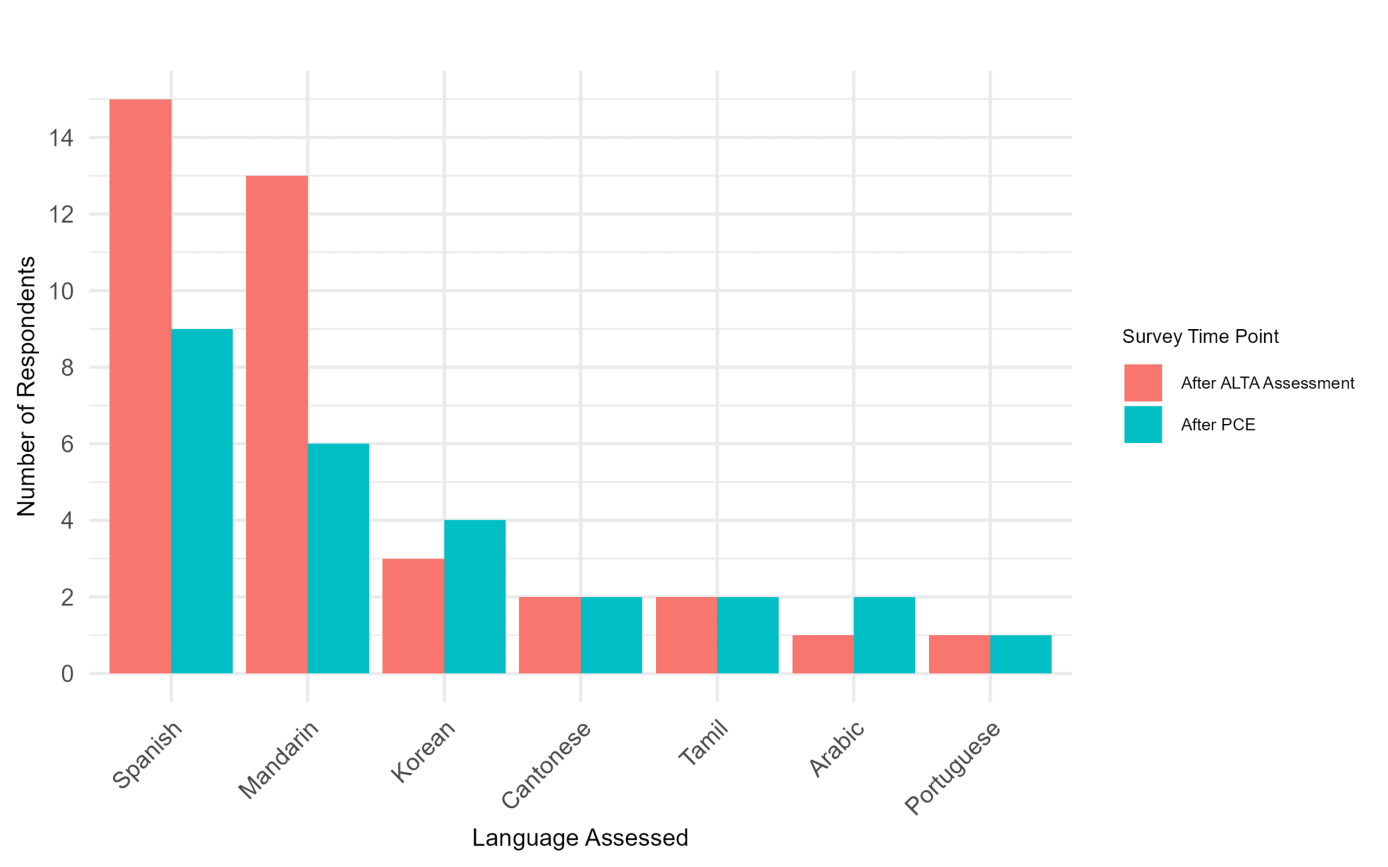
Participants’ Language Assessed by Survey Time Point PCE: Principal Clinical Experience

Of the 30 students who took the voluntary post-ALTA survey and had completed the PCE, 24 students responded about their experience using their non-English language with patients (Figure 1). Sixteen students (66.7%) reported being asked to interpret for others on their clinical teams, indicating frequent reliance on bilingual students as informal interpreters. Sixteen students (66.7%) reported never or rarely using the language button on clinical rotations. Half of the students (n=12, 50%) reported using their non-English language with patients multiple times per month or week, while four students (16.7%) used the language once per month.

The students who received the language button voluntarily reported positive experiences with wearing it in clinical rotations (Table 2). However, many students did not pick up the button for a variety of reasons related to the timing of clinical rotations. For example, students who took the ALTA exam in the PCE year were located at off-site affiliate hospitals for rotations, which posed a barrier for them returning to the medical school to pick up their language buttons. Some students took the ALTA exam in their final year after they had completed their clinical rotations, so they did not feel the need to pick up the language button.

In general, students reported positive language-concordant experiences with patients and positive clinical team experiences related to the students’ language skills (Table 2). However, many students were asked to interpret inappropriately; this was not best practice because the skills for direct communication and interpretation are distinct and require separate assessments. Furthermore, all information obtained by students needs to be independently verified by a supervising physician. The majority of students (14/16; 87.5%) who were asked to interpret agreed to do so, while only a few shared that they declined to interpret. These findings highlight the urgent need for institutional responsibility and faculty education to prevent the inappropriate reliance on trainees as interpreters by integrating the QMA into medical school training to formally recognize a student’s verified language status.

## Discussion

We demonstrated that the QMA is both feasible and acceptable among multilingual medical students. We observed substantial demand for the QMA, with 92 students signing up to take the exam primarily to receive a letter and button confirming their language qualification; notably, only students who completed the post-PCE survey received a $10 Starbucks gift card as a token of appreciation for their time. Overall, students valued the opportunity to have their non-English language skills formally verified for clinical care. We identified important gaps regarding setting expectations with both faculty and students regarding the purpose of the QMA for direct patient communication, not interpretation. However, there were important lessons learned in the implementation process that will guide future workflow improvements [6]. An overarching challenge was the variable use of the language buttons. Many students did not pick up the button, citing logistical barriers caused by the timing of clinical rotations. Despite this, students who did wear the buttons reported positive experiences, including building patient rapport and receiving appreciation from their clinical teams.

These findings underscore the critical need for robust faculty education and heightened institutional responsibility for non-English language proficiency assessments [11]. Clinical supervisors should be able to distinguish qualification for language-concordant care and professional interpretation [2]. To facilitate this, future directions should include the systematic integration of QMA into medical school curricula and clinical supervision policies [7]. Furthermore, incorporating a student's verified language proficiency into electronic health records would streamline expectations for qualified language-concordant care [4].

The study had several limitations. First, selection bias is inherent in the study design, as participation was limited to students who self-reported “very good” or “excellent” oral proficiency on the ILR scale. This recruitment strategy likely accounts for the high pass rate, and these results should be interpreted as representative of a highly proficient subset rather than the general medical student population [10].

Second, the survey design utilized anonymous responses to maximize participation rates. However, this approach precluded paired longitudinal analysis at the individual level [3]. While we used language as a linking variable to observe trends across time points, our findings are descriptive rather than inferential. Finally, we acknowledge that the ALTA Speaking and Listening test is not a gold standard for medical language proficiency, though there are no other products on the market that are recognized as such [5,8].

Additional research is needed to evaluate the impact of the QMA on student behaviors, team dynamics, and patient experiences and outcomes. Future educational efforts should focus on addressing these issues for both students and faculty, emphasizing that direct communication and medical interpretation are distinct competencies that require separate assessments.

## Conclusions

Multilingual medical students thought the QMA was feasible and acceptable. The formal recognition of oral proficiency appropriate for clinical communication is a valuable step forward for multilingual medical students who are uniquely positioned as both learners and contributors to direct patient care. Additional education is needed to distinguish the roles and skills between language-concordant communication and interpreter-mediated communication.

## Figures and Tables

**Figure 2:**
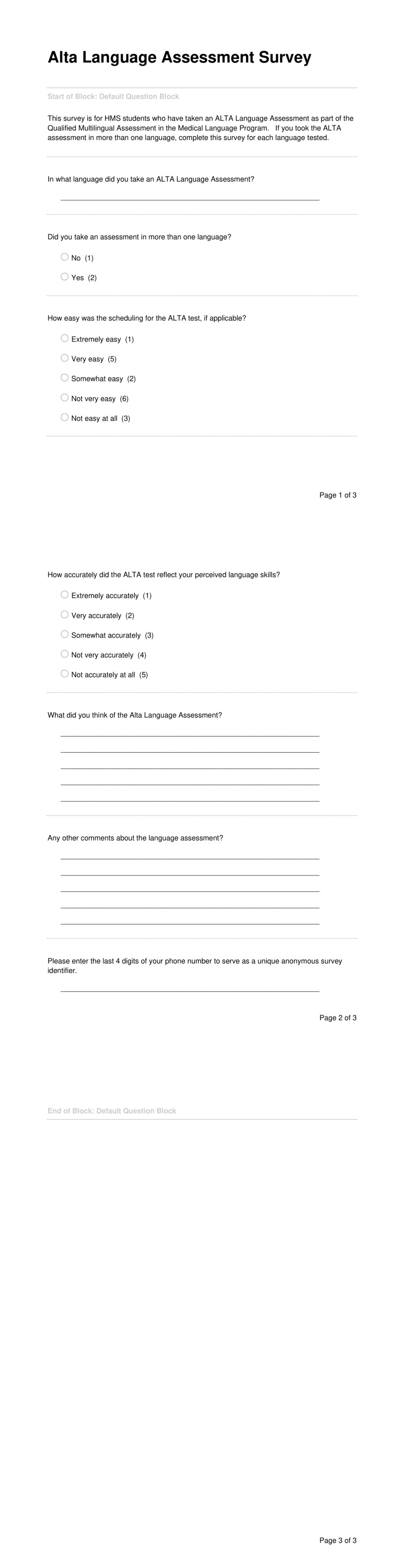
ALTA Language Assessment Survey

**Figure 3:**
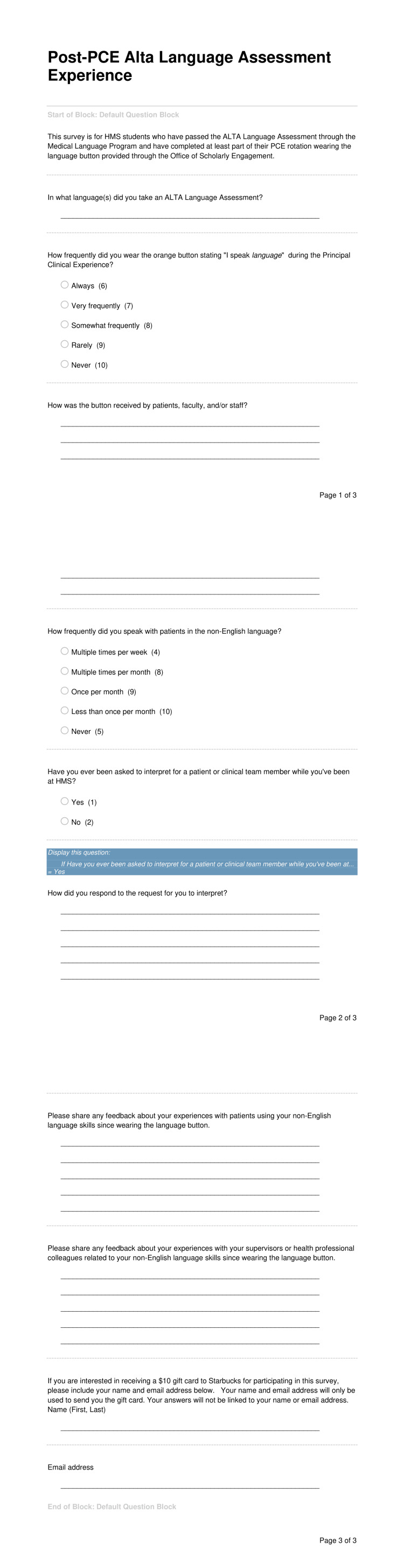
Post-PCE ALTA Language Assessment Experience Survey PCE: Principal Clinical Experience

**Figure 4:**
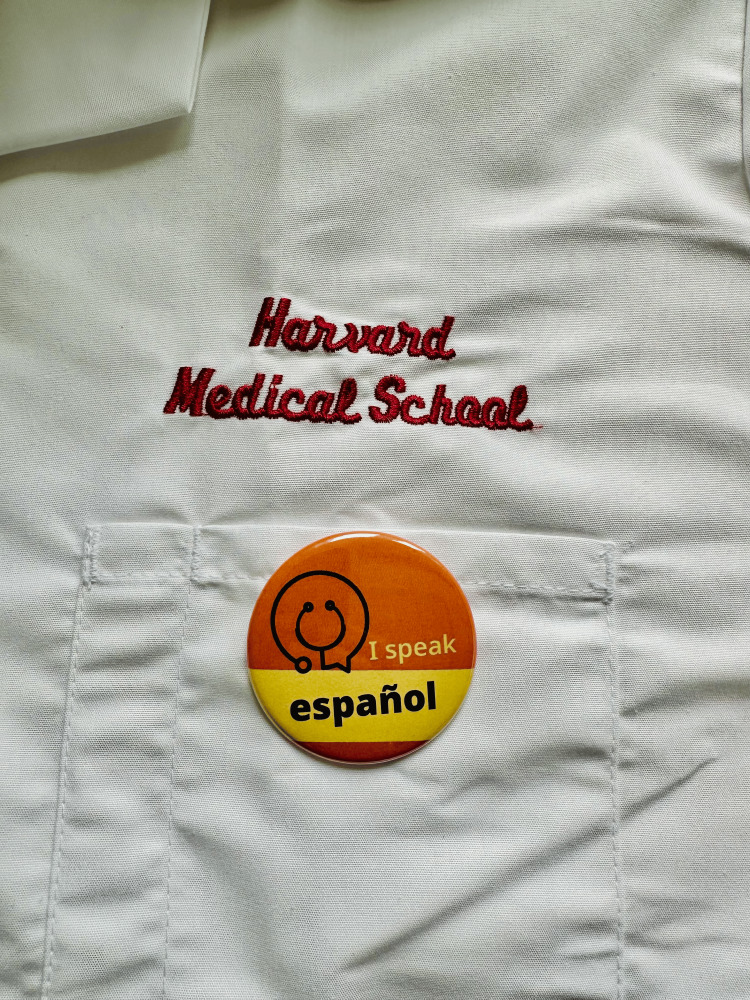
Qualified Multilingual Button for Medical Students Who Passed the ALTA Speaking and Listening Assessment
